# RNA-Seq transcriptome analysis of breast muscle in Pekin ducks supplemented with the dietary probiotic *Clostridium butyricum*

**DOI:** 10.1186/s12864-018-5261-1

**Published:** 2018-11-28

**Authors:** Yanhan Liu, Yaxiong Jia, Cun Liu, Limin Ding, Zhaofei Xia

**Affiliations:** 1 College of Veterinary Medicine, China Agricultural University, Beijing, 100193 China; 2 Institute of Animal Sciences, Chinese Academy of Agricultural Sciences, Beijing, 100193 China; 3 College of Animal Science and Technology, China Agricultural University, Beijing, 100193 China

**Keywords:** Breast muscle, *C. Butyricum*, De novo assembly, Pekin duck, RNA-Seq

## Abstract

**Background:**

Increased attention is being paid to breast muscle yield and meat quality in the duck breeding industry. Our previous report has demonstrated that dietary *Clostridium butyricum* (*C. butyricum*) can improve meat quality of Pekin ducks. However, the potential biological processes and molecular mechanisms that are modulated by dietary *C. butyricum* in the breast muscle of Pekin ducks remain unknown.

**Results:**

Supplementation with *C. butyricum* increased growth performance and meat yield. Therefore, we utilized de novo assembly methods to analyze the RNA-Seq transcriptome profiles in breast muscle to explore the differentially expressed genes between *C. butyricum*-treated and control Pekin ducks. A total of 1119 differentially expressed candidate genes were found of which 403 genes were significantly up-regulated and 716 genes were significantly down-regulated significantly. qRT-PCR analysis was used to confirm the accuracy of the of RNA-Seq results. GO annotations revealed potential genes, processes and pathways that may participate in meat quality and muscle development. KEGG pathway analysis showed that the differentially expressed genes participated in numerous pathways related to muscle development, including ECM-receptor interaction, the MAPK signaling pathway and the TNF signaling pathway.

**Conclusions:**

This study suggests that long-time dietary supplementation with *C. butyricum* can modulate muscle development and meat quality via altering the expression patterns of genes involved in crucial metabolic pathways. The findings presented here provide unique insights into the molecular mechanisms of muscle development in Pekin ducks in response to dietary *C. butyricum*.

**Electronic supplementary material:**

The online version of this article (10.1186/s12864-018-5261-1) contains supplementary material, which is available to authorized users.

## Background

Pekin duck is a popular poultry species worldwide because of its rapid growth and considerable breast muscle mass [1]. Duck meat, in particular breast meat, is considered a delicacy worldwide, especially in China. Breast muscle development of Pekin ducks usually begins at 4.5 and ends at 7 weeks of age [2]. However, to increase the economic gain, the market age of Pekin ducks can be advanced to 5 or 6 weeks by overfeeding [3]. If the quality of the breast muscle has not fully developed by this age, huge economic losses may result. It is therefore crucial to explore new approaches to improve the duck breast muscle yield and meat quality in practical production.

Diet modulation using additives such as oil, antioxidants and probiotics can improve muscle production, meat quality, and fatty acid composition. Many studies have demonstrated that dietary addition of probiotics such as *Lactobacillus plantarum* and *C. butyricum* can improve growth performance and meat quality in animals kept for meat [4, 5]. *C. butyricum*, a Gram-positive anaerobe, can generate butyric acid as well as form spores to aid survival in the intestine of healthy organisms [6]. Therefore, *C. butyricum* is a potential alternative to antibiotics to promote the growth rate and immune function of Cherry Valley ducks [7]. *C. butyricum* has been extensively applied in the poultry industry, because it has been shown to enhance growth performance, meat quality, and muscular fatty acid composition in broilers [8, 9]. The production and quality of breast meat are of extreme importance for consumers. Moreover, the profitability of the duck industry is mainly dependent on increasing the proportion of main segments in the carcass, especially the breast meat [10]. However, there has been insufficient research on the effects of *C. butyricum* in the breast muscle of Pekin ducks. Our previous work showed that appropriate supplementation (400 mg/kg) of *C. butyricum* can improve meat quality and lipid metabolism [11]. However, the underlying molecular regulatory mechanisms are still unknown.

Systems biology approaches are rapidly developing, and high-throughput transcriptome sequencing has been applied to explore the differentially expressed candidate genes that may be involved in particular biological processes. Simultaneously, Illumina sequencing technology allows more reliable gene identification [12, 13]. The relatively short reads generated by Illumina sequencing can be effectively de novo assembled and used for gene exploration and comparison of gene expression patterns [14]. Using a high-throughput RNA-Seq technique and secondary bioinformatics analysis, the differentially expressed genes (DEGs) in breast muscle were identified between Pekin ducks treated with *C. butyricum*-treated and a control group. This provided a basis for subsequent functional exploration of the DEGs in enhancing breast muscle development of Pekin ducks.

The aim of this study was to investigate the molecular mechanisms and the metabolic pathways involved in the regulation of the physiological changes induced by dietary *C. butyricum* which have the potential to enhance the efficiency of poultry production and meat quality, and to identify key genes involved in the promotion of duck muscle development of ducks. This work provides a theoretical basis for further study of the molecular mechanisms involved in Pekin duck muscle development. Elucidation of the underlying mechanisms could help us to seek novel approaches to optimize the use of probiotics in the poultry and livestock industries.

## Methods

### Ethics statement

The present animal study protocol was approved by China Agricultural University Ethics Committee (Permit No. CAU20170505–3) and closely followed the recommendations of the Guidelines for Experimental Animals. Before tissue sampling, all animals were humanely euthanized with sodium pentobarbitone (30 mg/kg BW) anesthesia. All efforts were made to minimize the suffering of animals.

### Experimental design, animals and management

Six hundred 1-day-old male Pekin ducks were obtained from a local commercial hatchery (Beijing Golden Star Duck Co., Ltd) and the experiment was carried out at the Experimental Center of China Agricultural University. The ducks were randomly allocated into two groups with five replicates of sixty ducks each, and raised in an air-conditioned room. The temperature was maintained at 32 to 34 °C from day 1 to 5, and then gradually reduced by 2 °C /week until it reached a final room temperature of between 23 and 25 °C. Ducks were allowed free access to feed and water with a 23 L: 1D lighting program during the experimental time. The groups were as follows: Control group, ducks were fed a corn-soybean basal diet; *C. butyricum*-treated group, ducks were given a basal diet supplemented with 400 mg/kg of *C. butyricum* (2.0 × 10^9^ CFU/g). The basal diet was formulated to meet or exceed the nutrient requirements according to the National Research Council (NRC, 1994) (Additional file 1: Table S1). The probiotic strain *C. butyricum* (Batch No. 20170325003) was obtained from Beijing Shine Biology Technology Co., Ltd., China. Feed intake was recorded by each replicate at 42 d of age. Ducks were weighed after feed deprivation for 6 h to calculate average body weight (ABW), average daily gain (ADG), average daily feed intake (ADFI), and the feed conversion ratio (FCR) at 42 d of age.

### Sample collection

At 42 d of age, twelve ducks per group were randomly selected and weighed. After dissection, the breast muscle was also weighed. Three muscle samples of each group were randomly selected and rapidly stored in liquid nitrogen for further analyses.

### RNA extraction and library preparation

Total RNA of the breast muscle tissues was extracted from the six randomly selected ducks using RNAiso puls kit (Takara, Osaka, Japan) following the manufacturer’s guidelines. After extraction, the concentration and purity of RNA were checked by an NanoDrop 2000 spectrophotometer (NanoDrop Technologies, Wilmington, DE) at 260 nm; and the integrity of the total RNA was measured by 1.2% agarose gel electrophoresis and an Agilent 2100 Bioanalyzer system (Agilent Technologies, Santa Clara, CA). The high-quality RNA samples for library construction were selected according to 260/280 nm ratio (1.9–2.1), RNA concentration (≥ 500 ng/μL) and RNA integrity number (RIN ≥ 8.0), respectively (Additional file 1: Table S2). Samples that did not satisfy the above quality criteria were re-extracted or not used for subsequent analysis.

Sequencing libraries were prepared by the TruSeq RNA Sample Prep Kit (Illumina, San Diego, CA) following the manufacturer’s guidelines. Briefly, the mRNA was purified from the total RNA using poly-T oligo-attached magnetic beads (Invitrogen, USA) to pull down the poly-A mRNA and cleaved into short fragments of about 300 bases. These fragmented mRNAs were then used as templates to synthesize cDNA by reverse transcriptase and random primers. The PCR was performed to amplify cDNAs to construct the library. After PCR enrichment, the quantity and quality of cDNA were measured by a NanoDrop 2000 spectrophotometer and Agilent 2100 Bioanalyzer. According to the manufacturer’s suggestions, 10 moll/μl Tris buffer (10 mmol Tris-HCl, 0.1% Tween 20, pH 8.5) was used to normalize the six cDNA libraries after construction. The cDNA libraries were sequenced on the Illumina sequencing platform (HiSeq 4000).

### Quality control and de novo transcriptome assembly

FastQC program (http://www.bioinformatics.babraham.ac.uk/projects/fastqc/) was used to check the quality of raw sequence reads generated from the Illumina Hiseq 4000 platform before alignment. Low-quality reads below a threshold quality of 20; reads shorter than 50 bp in length as well as reads containing adapter sequences, ploy-N and the sequencing primer from the raw data were removed to produce clean reads [15]. Meanwhile, Error %, Q30, GC-content % and sequence duplication level of the clean data were evaluated (Additional file 1: Table S3). All of the subsequent analyses and annotation were depended on high quality clean reads.

Raw reads were dealt with to filter redundant sequences, low-quality reads and artificial repeats to obtain clean reads by SeqPrep software (https://github.com/jstjohn/SeqPrep) and Sickle software (https://github.com/najoshi/sickle) [16]. The first assembly was performed using clean data by SeqPrep software to generate a de novo dataset [17] . The contiguous nucleotide sequences (contigs) were further extended by combining the de novo dataset with all available duck mRNA sequences from Ensembl, and then, the sequences were assembled by Trinity software(http://trinityrnaseq.github.io) [18]. At last only unique contigs ≥300 bp in length can be used for downstream study after assembly.

### Annotation and classification of the de novo assembled transcriptome

BLASTX (https://blast.ncbi.nlm.nih.gov/Blast.cgi) search (*E*-value <1e^− 5^) was conducted to align the de novo assembled transcriptome against four databases (NR-Non-Redundant Protein Sequence Database, GO-Gene Ontology, KEGG-Kyoto Encyclopedia of Genes and Genomes, and COG-Clusters of Orthologous Groups) to obtain the annotation and classification information to predict the biological function.

### GO annotation and KEGG pathway analysis of differentially expressed genes

The differentially expressed genes (DEGs) between *C. butyricum*-treated group and control group were identified using edgeR software (http://www.bioconductor.org/packages/2.12/bioc/html/edgeR.html). Genes with a *P*-value < 0.05 and fold-change *≥*2 or ≤ 0.5 were considered significant. We used Goatools software(https://github.com/tanghaibao/GOatools)to evaluate DEGs between the control group and *C. butyricum-*treated group by GO analysis [19, 20]. DEGs at *P*-value < 0.05 and fold-change ≥2 or ≤ 0.5 were regarded as significantly enriched. Kyoto Encyclopedia of Genes and Genomes (KEGG) (http://www.genome.jp/kegg/) is a public database resource which was used to predict the potential metabolic pathways or signal transduction pathways of DEGs. KOBAS software 3.0 (http://kobas.cbi.pku.edu.cn/index.php) was used to evaluate the statistical enrichment of DEGs among KEGG pathways (Corrected *P*- value < 0.05).

### qRT-PCR confirmation of differentially expressed genes

To confirm the reproducibility and repeatability of the differential expressed genes obtained from RNA-Seq data, we performed qRT-PCR to evaluate 10 randomly selected genes related to muscle development. Total RNA was extracted using TRIzol reagent (Invitrogen) and was reverse-transcribed using the TransScript One-Step gDNA Removal and cDNA Synthesis SuperMix (TransGen Biotech, Beijing, China) according to the manufacturer’s guidelines. Specific primers of each gene were designed using primer premier 5.0 were synthesized by Sunbio Biotech Ltd. (Beijing, China) (Table 1). The Top Green qPCR SuperMix (TransGen Biotech, Beijing, China) was used to perform qRT-PCR procedure following the manufacturer’s instruction. The reaction mixtures were added in a 96-well plate at 95 °C for 30 s, followed by 40 cycles of 95 °C for 5 s and 55 °C for 34 s, and then followed by a melting curve using an ABI QuantStudio 7 Flex Sequence Detection System (Applied Biosystems, Foster City, CA). The glyceraldehyde-3-phosphate dehydrogenase (*GAPDH*) gene of *Anas platyrhynchos* was set as an endogenous reference gene. After each real-time experiment, dissociation curve analysis was conducted to make sure that only one product was amplified. All experiments were preformed in triplicates for each biological repeat.

**Table 1:** Primers information used for qTR-PCR in this study

Gene	Primer sequence (5′ → 3′)	Gene ID	Ampicon size (bp)
MGP-F	GGGAGATCTGCGAAGACTACTA	NW_004677540	98
MGP-R	CTTAGTCCTCCTCCTCCCATAA		
FLNC-F	CCTACTCTACCTGGGAACTACA	NW_004677877	106
FLNC-R	GGGACCGTCTTCTGTTATCATC		
FBXO_25-F	ACCCAATAAAGGAGCAGTATGG	NW_004676507	107
FBXO_25-R	GTGTCCTCCATCCTTGAGTAAC		
LAMB4-F	GCTGCAAGTCTGGAGGATAAA	NW_004676386	126
LAMB4-R	CAAGTGCCTCCAGTGAAAGA		
MGAT3-F	GGCTTTCGGCAGTATGAGAA	NW_004678325	134
MGAT3-R	GCTGACACCAGTTTGAAGTAGA		
MYL3-F	TGTTCGACAAGGAGGGAAATG	NW_004677386	120
MYL3-R	CATCTTCCTGACCAGCCATTAG		
TPM3-F	GCTGGTAGAGGAGGAGTTAGA	NW_004679752	103
TPM3-R	CATGCCTCTTTCGCTTTCATC		
PPP1R3B-F	CAGACTCAAAGGAGAGGTCAAG	NW_004676586	101
PPP1R3B-R	TCAATCCCACCCAAACTCTG		
DGKD-F	CTGAAGAGGGCAAGGAGTTAAG	NW_004678870	96
DGKD-R	TCCGTTGGTTCCTCACTTTG		
HSPB1-F	CAATGTTGCCAAGCCTGATCTA	NW_004676844	171
HSPB1-R	GCATCCAGTTCAATCCACGAGTT		
GAPDH-F	GGTAGTGAAGGCTGCTGCTGATG	NW_004676785	197
GAPDH-R	CCACCACACGGTTGCTGTATCC		

*MGP* Matrix Gla protein, *FLNC* Filamin C, *FBXO_25* F_box protein 25, *LAMB4* Laminin subunit beta 4, *MGAT3* Mannosyl (beta-1,4-)-glycoprotein beta-1,4-N-acetylglucosaminyltransferase, *MYL3* Myosin light chain 3, *TPM3* Tropomyosin 3, *PPP1R3B* Protein phosphatase 1 regulatory subunit 3B, *DGKD* Diacylglycerol kinase delta, *HSPB1* Heat shock protein family B member 1, *GAPDH* Glyceraldehyde-3- phosphate dehydrogenase

### Statistical analysis

Data are presented as mean ± S.D. Comparison between *C. butyricum*-treated group and control group of growth performance and meat quality data was performed using the univariate ANOVA procedure with SPSS19.0 software (SPSS Inc., Chicago, IL, USA). Relative fold changes of target gene expression was calculated by the 2^-ΔΔCt^ method [21]. The ΔCt value was confirmed by subtracting the target Ct of each sample from its *GAPDH* Ct value.

## Results

### Effects of *C. butyricum* on performance and meat quality of Pekin ducks

During the experimental period (42 days), in comparison with the control group, increased ABW, average daily gain (ADG), feed conversion ratio (FCR), and percentage of breast muscle as well as decreased average daily feed intake (ADFI) were observed significantly (*P* < 0.05) in the *C. butyricum*-treated group (Table 2). Moreover, no significant differences were found for pH_24h,_ the lightness and the yellowness of meat color, whereas increased pH_45min_, higher redness of meat color and lower drip loss and shear force of breast muscle in ducks were measured significantly (*P* < 0.05) between the *C. butyricum*-treated group and the control group (Additional file 1: Table S4).

**Table 2:** Effects of *Clostridium butyricum* on growth performance of Pekin ducks at 42d

Parameters	Control	Treatment
AMB (kg)	2.96 ± 0.07^a^	3.20 ± 0.07^b^
ADG (g/d)	77.4 ± 0.17^a^	81.8 ± 0.25^b^
ADFI (g/d)	152.00 ± 1.00^a^	144.00 ± 2.65^b^
FCR (g/g)	1.96 ± 0.01^a^	1.76 ± 0.03^b^
Breast muscle (%)	3.76 ± 0.28^a^	4.24 ± 0.01^b^

Values are mean ± SD of 12 independent determinations;

Means with different superscript letters a, b indicate that there are significant differences (*P <* 0.05) between two groups in the same row

*ABW* Average body weight, *ADG* Average daily gain, *ADFI* Average daily feed intake, *FCR* Feed conversion ratio

### De novo transcriptome assembly profiles evaluation

The summary information of the quality and alignment of transcriptome sequence of the six breast muscle samples were presented in Table 3. Approximately 6.0 Gb high-quality clean data per sample were obtained for further analysis after removing low-quality reads, cutting adapter, and filtering. The duck genome sequence had been released; however, the low quality of the assembled genome and annotations made the transcriptome analysis difficult. In this study, only approximately 55% of clean reads were mapped onto the reference duck genome (http://asia.ensembl.org/Anas_platyrhynchos/Info/Index?db=core) with varied alignments for our data (Additional file 1: Table S5). Thus, Trinity software was used for de novo assembly with the clean reads; more than 86.60% of clean reads per specimen can be mapped back to the reference transcript after alignment (Table 3, Additional file 1: Table S6). Almost 68.15–72.98% clean reads were aligned in a unique manner, while 15.53–18.46% as multiple-mapped reads. The CG% of each sample was all above 50% (Additional file 1: Table S6). A total of 73,740 unigenes sequences were de novo assembled with an N50 length of 2192 bp. After removing low-quality sequences and redundancies, the de novo transcript had 91,365 sequences with an N50 length of 2733 bp. The length of transcripts ranged from 201 to 61,746 bp. A comparison of the duck transcripts to the best candidate assembly which could be used as a reference was presented in Table 4. Our de novo assembled duck transcripts could be better performed assembly, increasing the mapping rate of over 86% per muscle sample. Thus, comparison among the three datasets revealed that our de novo assembled duck transcripts were better to be used to explore gene expression patterns instead of the reference duck genome.

**Table 3:** Summary statistics for sequence quality and alignment information of six breast muscle samples in two groups

Sample	A1	A2	A3	B1	B2	B3
Group	Control	Control	Control	Treatment	Treatment	Treatment
Raw reads	61,522,238	66,734,468	55,944,188	56,658,102	52,848,740	63,579,662
Raw bases	9.29E+ 09	1.01E+ 10	8.45E+ 09	8.56E+ 09	7.98E+ 09	9.6E+ 09
Clean reads	59,695,558	64,690,588	54,170,296	54,959,360	51,137,356	61,709,484
Clean bases	8.83E+ 09	9.57E+ 09	8.01E+ 09	8.14E+ 09	7.55E+ 09	9.13E+ 09
Error%	0.0123	0.0123	0.0126	0.0123	0.0124	0.0122
Q30 (%)	94.91	94.84	94.52	94.82	94.67	94.99
GC contents (%)	55.00	53.92	55.35	53.95	55.94	54.66
Total mapped reads	51,698,314	56,863,042	47,906,218	48,284,460	45,259,894	54,291,950
Uniquely mapped reads	40,680,290	45,051,062	39,062,890	38,983,440	37,319,098	43,728,402
Multiple mapped reads	11,018,024	11,811,980	8,843,328	9,301,020	7,940,796	10,563,548
Mapping rate (%)	86.60	87.90	88.44	87.85	88.51	87.98

**Table 4:** Characteristics of the Pekin duck gene de novo assembly

Type	Unigene	Transcripts
Total sequence number	73,740	91,365
Total sequence (bp)	83,369,834	125,267,765
GC content	47.92%	48.20%
Largest (bp)	61,746	61,746
Smallest (bp)	201	201
Average (bp)	1130.59	1371.07
N50	2192	2733
N90	432	574

### Functional annotation and classification

Homology analysis of the obtained sequences after clustering was performed by BLASTX (https://blast.ncbi.nlm.nih.gov/Blast.cgi) against NR databases at NCBI. The distribution of top hit species were determined and the majority of the annotated sequences were consistent with known nucleotide sequences of bird species, with *Anas platyrhynchos* (4604) followed by *Gallus gallus* (1620) (Additional file 2: Fig. S1). Generally speaking, nearly all sequences of Pekin duck should be similar with *Anas platyrhynchos*, while the sequences information of *Anas platyrhynchos* is not complete in the NCBI.

**Fig. 1:**
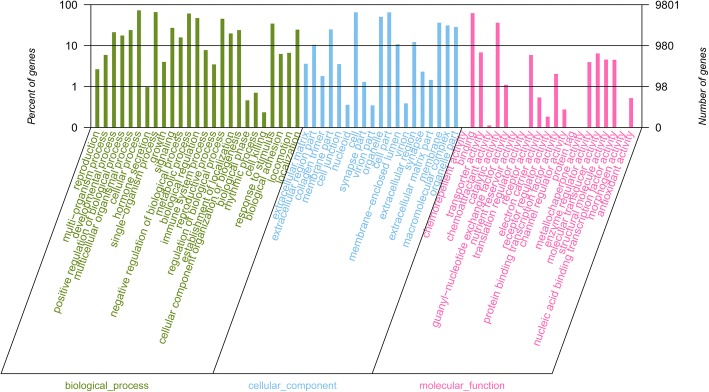
Annotation of genes using Gene ontology in breast muscle of Pekin duck. The number of genes for each GO annotation is exhibited in right axis, and the proportion of genes for each GO annotation is listed in left axis

**Fig. 2:**
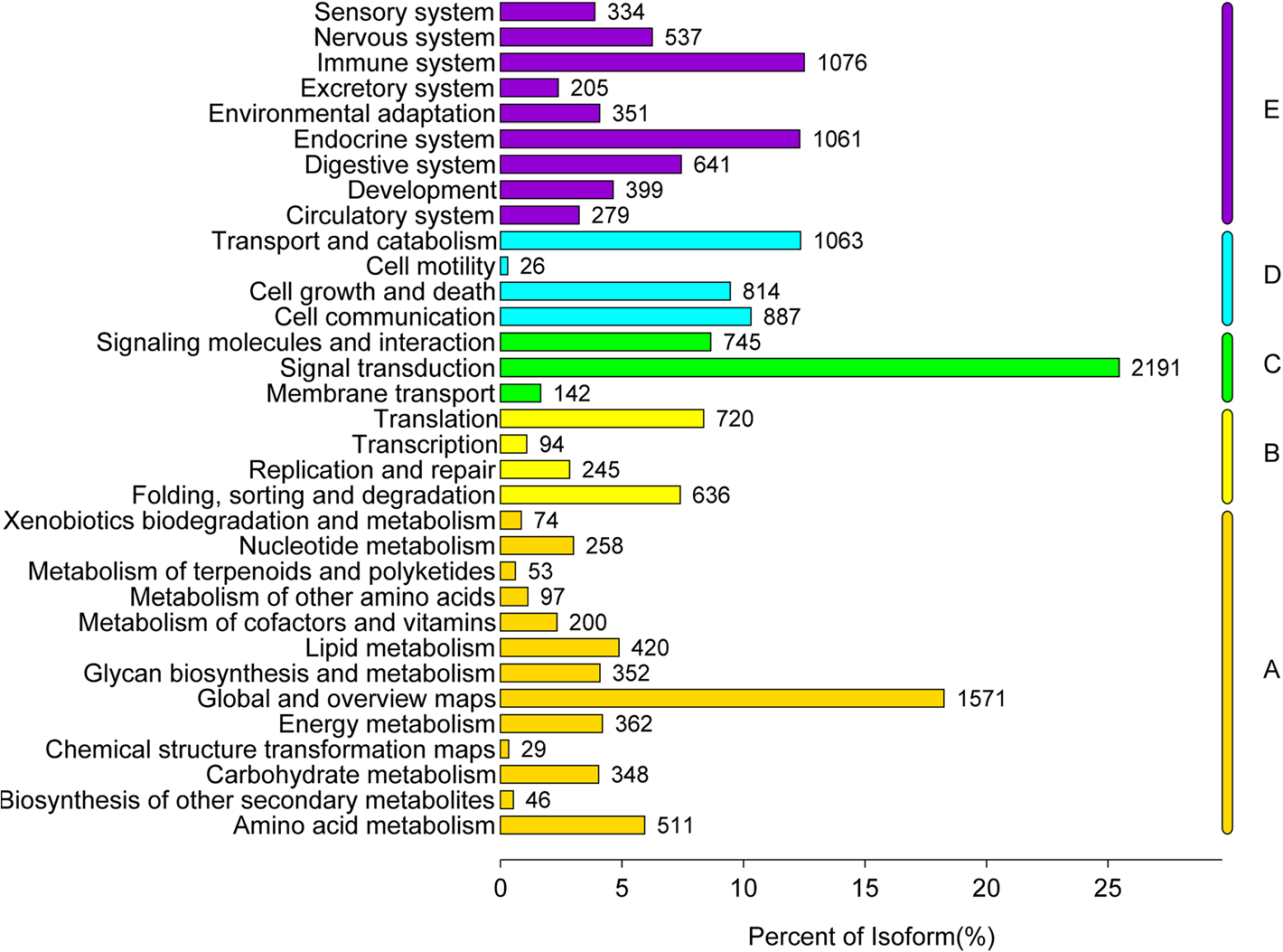
KEGG pathway classified annotation of genes in breast muscle of Pekin duck. **a** Metabolism; **b** Genetic Information Processing; **c** Environmental Information Processing; **d** Cellular Processes; **e** Organismal Systems

**Fig. 3:**
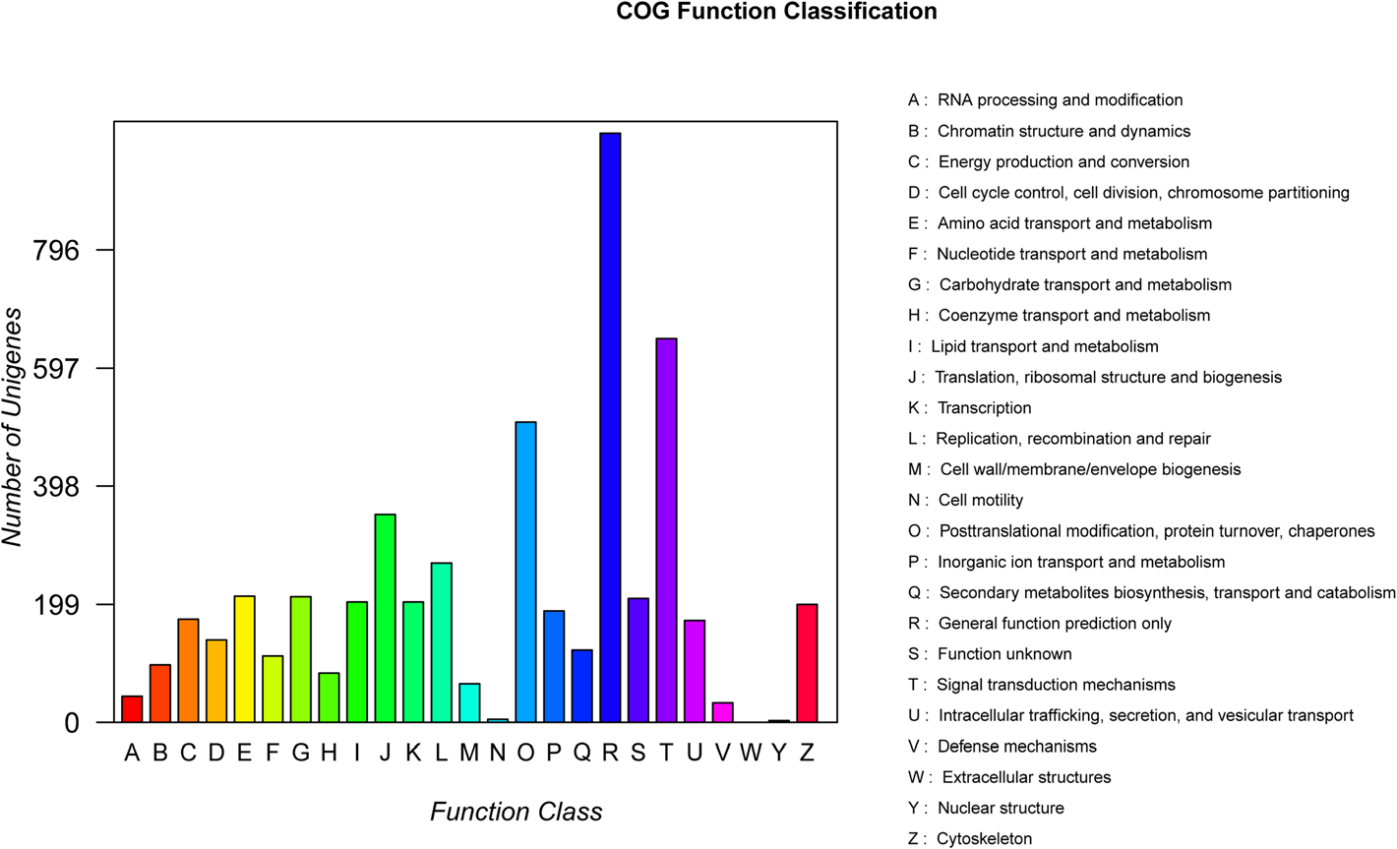
COG function classification of genes in breast muscle of Pekin duck

A total of 73,740 genes were allocated to three primary GO categories (biological_process, cellular_component, and molecular_function), which were subsequent assigned to 65 functional terms. The total genes were mainly related to cellular process (7104), single-organism process (6452), metabolic process (5941), cell (6346), cell part (6345), binding (6083), and catalytic activity (3543) (Fig. 1, Additional file 2: Fig. S2). According to the KEGG analysis, the top 5 classifications of genes were: signal transduction, global and overview maps, immune system, endocrine system transport and catabolism, respectively (Fig. 2). The top 20 enriched pathways included “PI3K-Akt signaling pathway”, “Focal adhesion”, “Regulation of actin cytoskeleton”, “MAPK signaling pathway”, and “Protein processing in endoplasmic reticulum” which may be associated with muscle development (Additional file 2: Fig. S3). In addition to GO analysis, COG analysis was preformed to deeply identify the function of the assembled unigenes. Genes were annotated based on the Cluster of COG database (Fig. 3). The largest number of matched genes was associated with signal transduction mechanism which is corresponded with KEGG pathways annotation.

### Analysis of differential expressed genes

Gene expression levels were defined using fragments per kilobase of transcript per million mapped reads (FPKM), which is proportional to the quantity of cDNA fragments derived from the gene transcripts. The lowest limit of gene expression level is set as 0.5 FPKM in at least one of the samples. Based on the limit, a total of 56,797 genes were confirmed to express in the breast muscle tissues. The correlation analysis according to the gene expression patterns revealed that the correlations of six samples were greater than 0.80 (Additional file 3).

**Fig. 4:**
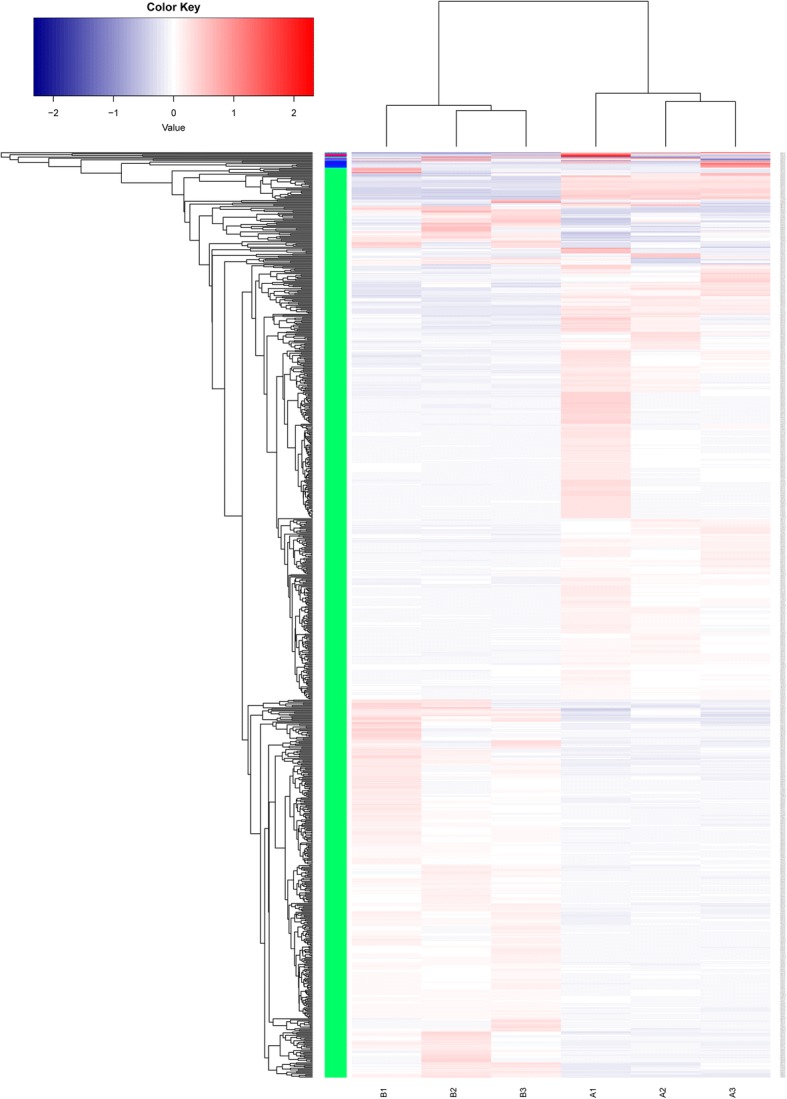
Cluster analysis of DEGs in breast muscle between control group (A1, A2, and A3) and treatment group (B1, B2, and B3). Values of log_10_FPKM were conducted at normalized transformation before clustering. Red indicates high expressed genes, and green indicates low expressed genes. Each column represented a sample, and each row represented a gene. The left was the tree diagram of gene clustering, and the right was the name of each gene. The closer the two gene branches were, the closer their expression level was. The upper part was the tree diagram of sample clustering, and the bottom was the name of each sample. The closer the two sample branches were to each other, the closer the expression pattern of all genes in the two samples was and the closer the change trend of gene expression quantity was

**Fig. 5:**
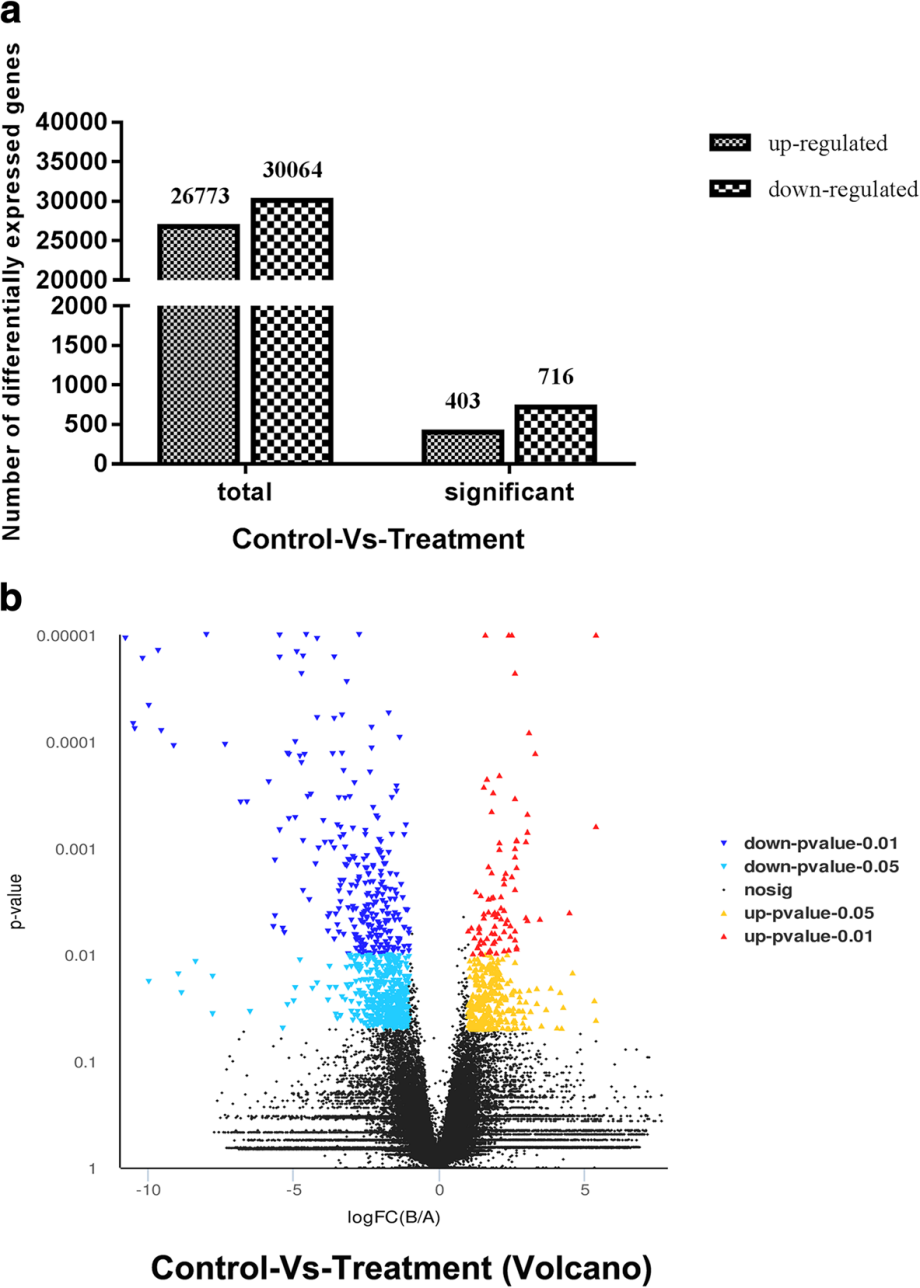
**a** The numbers of differentially expressed genes (DEGs; up/down-regulated) in breast muscle between control group and treatment group. **b** Volcano plot of global DEGs in breast muscle between control group and treatment group. Red dots (Up) represent significantly up-regulated genes (*P* < 0.05, fold change ≥2); Yellow dots (Up) represent extremely significantly up-regulated genes (*P* < 0.01, fold change ≥2); Mazarine dots (Down) represent significantly down-regulated genes (*P* < 0.05, fold change ≤0.5); Wathet dots (Down) represent extremely significantly down-regulated genes (*P* < 0.01, fold change ≤0.5); black dots (nosig) represent insignificantly differential expressed genes

A total of 1119 genes were found expressed differentially at a fold-change ≥2 or ≤ 0.5 at *P-* value < 0.05 in response to *C. butyricum* treatment. Cluster pattern analysis of DEGs between control group (A1, A2, and A3) and the *C. butyricum*-treated group (B1, B2, and B3) was listed in Fig. 4, indicating that DEGs of each three samples selected from the control group or *C. butyricum*-treated group presented the approximately similar expression pattern. Among these genes, 403 out of 26,733 genes were up-regulated significantly while 716 out of 30,064 genes were down-regulated significantly (Fig. 5). The fold changes induced by dietary supplementation with *C. butyricum* ranged from − 10.77 to 5.41 (Additional file 3). Compared to the control group, a total of 6 up-regulated genes and 6 down-regulated genes in *C. butyricum*-treated group which have orthologs in *Anas platyrhynchos* gene database and may be associated with muscle development were shown in Table 5.

**Table 5:** The up- and down-regulated Genes associated with breast muscle development

Sequence ID	Gene symbol	Control_A (FPKM)	Treatment_B (FPKM)	Fold Change (log_10_(B/A))	*P-*value	FDR
Up-regulated genes						
c4419_g1	MGP	229.5	525.36	1.19	2.96E-02	1.00E-00
c68736_g3	FLNC	2.83	6.89	1.25	1.41E-02	1.00E-00
c70251_g1	FBXO_25	6.52	19.49	1.56	2.67E-04	3.03E-01
c4293_g1	LAMB4	0.05	0.30	1.43	3.33E-02	1.00E-00
c109044_g1	MGAT3	0.03	5.56	5.39	6.89E-10	1.96E-05
c69956_g1	ASIC4	1.89	5.43	1.47	9.85E-03	1.00E-00
Down-regulated genes						
c56839_g3	MYL3	182.82	0.06	−10.2	1.66E-05	5.90E-02
c56924_g1	TPM3	3.62	0.05	−4.66	8.53E-04	5.53E-01
c53045_g1	PPP1R3B	4.01	0.61	−2.53	4.36E-02	1.00E-00
c64135_g1	DGKD	0.48	0.19	−1.01	3.77E-02	1.00E-00
c61682_g1	HSPB1	1549.43	546.55	−1.46	1.19E-02	1.00E-00
c54755_g1	HSPB7	279.72	22.95	−3.60	6.10E-05	1.45E-01

*MGP* Matrix Gla protein, *FLNC* Filamin C, *FBXO_25* F_box protein 25, *LAMB4* Laminin subunit beta 4, *MGAT3* Mannosyl (beta-1,4-)-glycoprotein beta-1,4-N-acetylglucosaminyltransferase, *ASIC4* Acid sensing ion channel subunit family member 4, *MYL3* Myosin light chain 3, *TPM3* tropomyosin 3, *PPP1R3B* Protein phosphatase 1 regulatory subunit 3B, *DGKD* Diacylglycerol kinase delta, *HSPB1* Heat shock protein family B member 1, *HSPB7* Heat shock protein family B member 1, *FPKM* Fragments per kilobase of transcript per million mapped reads

### GO annotations and KEGG pathway analysis of differential expressed genes

**Fig. 6:**
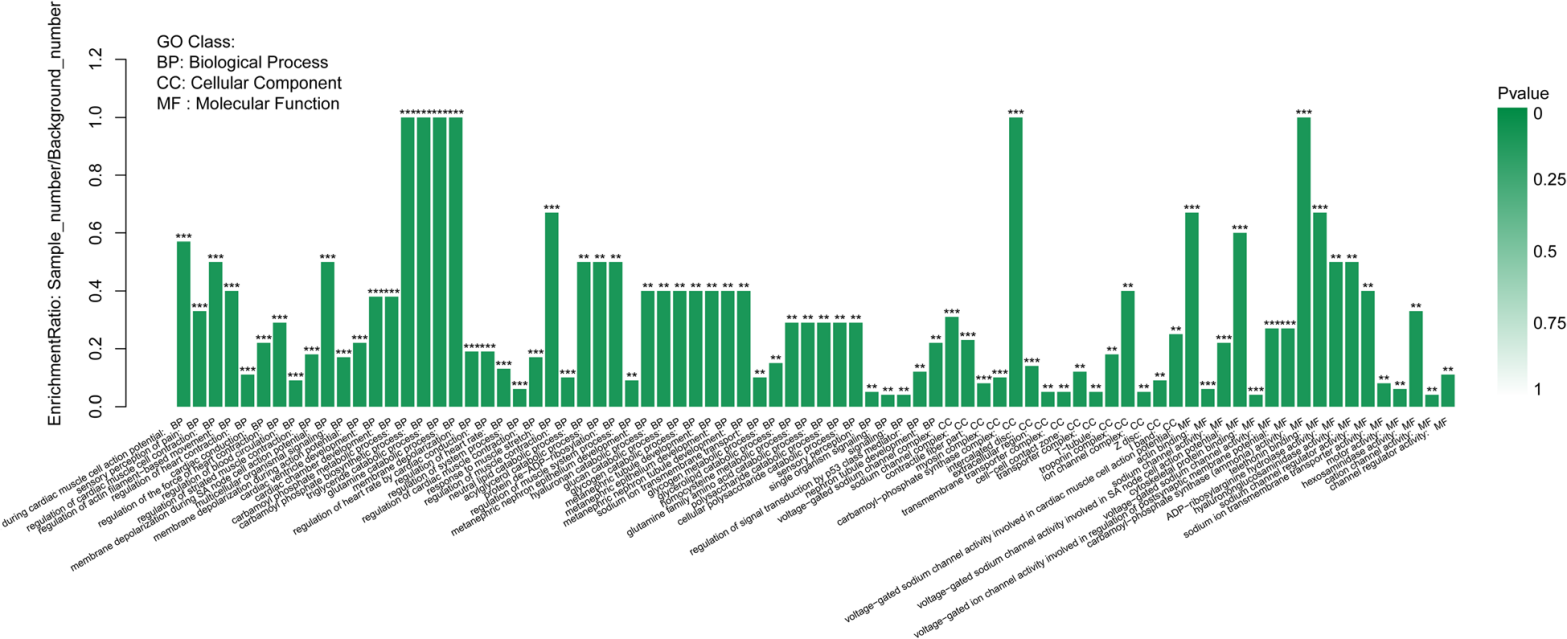
Gene ontology (GO) annotation of DEGs between control and treatment group. * means GO categories with significant enrichment

**Fig. 7:**
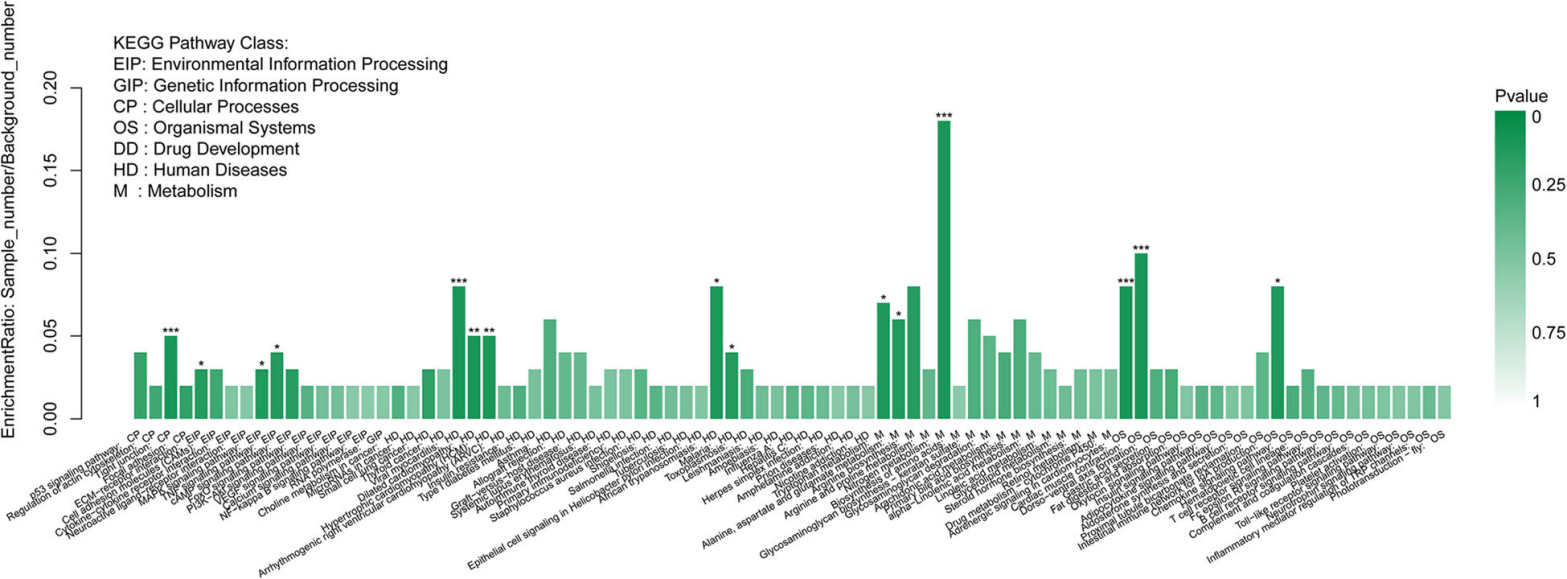
KEGG Pathway analysis of DEGs between control group and treatment group. * means KEGG pathway with significant enrichment

Gene Ontology (GO) enrichment analysis was done according to three categories including biological process, cellular component, and molecular function. Of these categories, most DEGs were enriched in the “biological process” category (Fig. 6, Additional file 2: Fig. S4, Additional file 4). Within the “biological process” category, “regulation of striated muscle contraction”, “regulation of muscle system process”, “regulation of actin filament-based process”, “muscle tissue morphogenesis”, and “triglyceride catabolic process” were the most primary subcategories. As for the “cellular component” category, most DEGs were assigned to “contractile fiber part”, and “myosin complex”. Within the “molecular function” category, the mainly enriched subcategories were “metal ion transmembrane transporter activity”, “cation channel activity”, “passive transmembrane transporter activity”, and “substrate-specific channel activity” (*P <* 0.05). The following genes participate in above GO terms: *MYH6, SCN5A, TBX1, MYOZ2, AQP1, KCNC4, MYL3, CHRNA1, MGP, HSPB1, SCN4B, KCNQ5, ATP2A, CPS1, TPM3, TNNI1, FLNC, ASIC4, FBXO_25,* and *LAMB4* (Additional file 3). After that, the DEGs were annotated through KEGG to confirm enriched pathways. The significantly (Corrected *P <* 0.05) enriched pathways included “Tight junction”, “ECM-receptor interaction”, “MAPK signaling pathway”, “TNF signaling pathway”, “Nitrogen metabolism”, and “Alanine, aspartate and glutamate metabolism” in our study (Fig. 7, Additional file 5).

### Confirmation of gene expression with quantitative RT-PCR

**Fig. 8:**
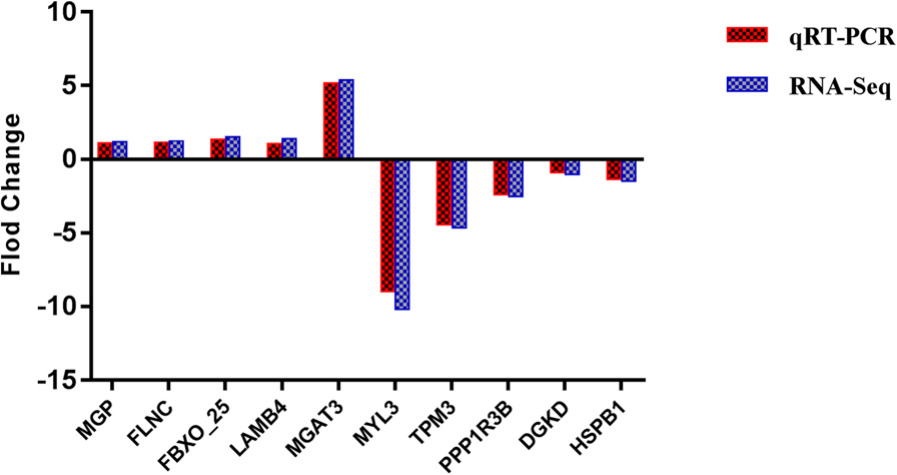
Expression levels of selected DEGs quantified by quantitative reverse transcription-PCR (qRT-PCR). *GAPDH* was used as an internal control, and data are presented as fold change (*N* = 6 per group)

To confirm the differentially expressed genes of breast muscle between control and treatment groups obtained by RNA-Seq, ten differential expressed genes (*MGP, FLNC, FBXO_25, LAMB4, MGAT3, MYL3, TPM3, PPP1R3B, HSPB1,* and *DGKD*) were randomly selected and their expression patterns were quantified by qRT-PCR. *MGP, FLNC, FBXO_25, LAMB4,* and *MGAT3* were up-regulated genes and *MYL3, TPM3, PPP1R3B, HSPB1,* and *DGKD* were down-regulated genes in high throughput RNA-Seq. The qRT-PCR results showed the similar down- or up-regulated trend in the expression of these genes (Fig. 8); therefore, the qRT-PCR expressions validate the findings by high throughput RNA-Seq.

## Discussion

There has recently been great interest in achieving higher growth performance and better meat quality in livestock [22]. Increasing attention is being paid to the nutritional levels and health benefits of meat, especially Pekin duck meat [23]. In this study, increased ABW and ADG and decreased FCR indicated that *C. butyricum* could feasibly be used to replace antibiotics to promote the growth of Pekin ducks. *C. butyricum* has beneficial effects on the promotion of growth, including enhanced immune function, improved meat quality, and balanced intestinal microflora [24–26]. The yield of breast muscle, the main source of Beijing roast duck, is very important [11]. In the present study, the percentage of breast muscle increased significantly. This showed that long-term dietary supplementation of *C. butyricum* alters metabolic pathways in muscle, which leads to the increased breast meat yield and meat quality [27].

Muscle development is a complicated physiological process including cell regeneration, differentiation, and migration. The underlying molecular mechanisms have been elucidated by high-throughput transcriptome analysis; however, the reference duck genome is incomplete, influencing the accuracy and precision of further analysis. In one recent study, only approximately 50% of reads could be mapped to the reference duck genome [28], which is consistent with difficulties we experienced. Therefore de novo assembly of the duck transcriptome was performed. Based on KEGG and COG classifications, many unigenes were enriched in signal transduction, indicating the importance of signal regulation in muscle development. Meanwhile, many DEGs enriched in the regulation of muscle system processes, muscle tissue morphogenesis, regulation of actin filament-based process, myosin complex, triglyceride catabolic process, and signaling pathways related to signal transduction were identified, indicating that muscle development requires a great deal of protein synthesis and signal transduction [29]. The DEGs identified by RNA-Seq were confirmed by qRT-PCR.

Myogenesis is an extremely complicated physiological process comprising myogenic progenitor proliferation, myoblast proliferation and differentiation, laeding to mature muscle [30]. Myogenesis can be regulated by many genes and pathways. We observed the up-regulation of genes related to myogenesis in the breast muscle of Pekin ducks treated with dietary *C. butyricum*. *MGP* (Matrix Gla protein) expression was increased in the *C. butyricum*-treated group. *MGP* shows an up-regulated expression pattern early in muscle cell development, suggesting that this high expression could be associated with cell differentiation and proliferation [31, 32]. Filamins are a family of actin binding proteins that are specifically expressed in skeletal muscle and were first identified by their ability to crosslink actin filaments [33]. Filamin C (*FLNC*), which is mainly expressed in muscle cells, interacts with myotilin at the Z-band edge [34] and is required for the maintenance of the structural integrity of skeletal muscles [35]. The high expression level in breast muscle cells in the *C. butyricum*-treated group suggests that *FLNC* may be a potential regulator facilitating muscle development. *FBXO_25* (F_box protein 25) also showed increased expression between the *C. butyricum*-treated and control group. This is a member of the MAPK pathway that regulates numerous biological processes including cell growth, differentiation and apoptosis in skeletal muscle development [36]. *FBXO_25* may facilitate muscle development in the *C. butyricum*-treated group by specifically activating the MAPK signaling pathway. The *LAMB4* (laminin subunit beta 4) is differentially expressed during pig muscle development, and has been proposed to be a positive factor that can activate myofiber formation [29], hence the elevated expression of *LAMB4* observed in the *C. butyricum*-treated group in our study is likely to contribute to the activation of myofiber formation in duck muscle. *MGAT3* also showed increased expression in the *C. butyricum*-treated group. Triacylglycerol synthesis is a critical function in muscular physiological processes, and *MGAT3* (mannosyl (beta-1,4-)-glycoprotein beta-1,4-N-acetylglucosaminyltransferase) can catalyze the synthesis of diacylglycerol from monoacylglycerol, a key step in improving meat quality and fatty acid composition [37]. These up-regulated genes work together to promote the muscle development of *C. butyricum*-treated Pekin ducks.

The expression levels of some genes were downregulated in the *C. butyricum*-treated Pekin ducks. *MYL3* (Myosin light chain 3) combines calcium ions, promotes muscle development and participates in the contraction of cardiac muscles. *MYL3/2/11* and *TNNI1/2/3* (troponin I type 1/2/3 (skeletal, slow)) are also candidate genes in chicken embryonic muscle development [38]. However, *MYL3* is not expressed during the myogenesis process in vitro or in mature skeletal muscles [39]. Moreover, *MYL3* was down-regulated in pork with high drip loss, which is the ability of the skeletal muscle to maintain water post-mortem [40]. The down-regulated expression of *MYL3* in the *C. butyricum*-treated group in our study confirmed these previous observations. *TPM3* (tropomyosin 3) is associated with nemaline myopathy, which presents as typically type 1 fiber hypotrophy, and mutations in the *TPM3* gene can induce congenital fiber-type disproportion, which is characterized by generalized muscle weakness [41, 42]. The down-regulated expression of *TPM3* in the *C. butyricum*-treated group is therefore beneficial for muscle development. *PPP1R3B* (Protein phosphatase 1, regulatory (inhibitor) subunit 3B) is a promising functional candidate gene for muscle development or meat quality [43, 44]. Expression of the *PPP1R3B* gene is negatively associated with pH_24h_ of muscle in pigs [45], and higher expression is seen in pork with low muscle pH in agreement with our results. This indicates that lower expression of *PPP1R3B* could contribute to the increased post-mortem muscle pH in the *C. butyricum*-treated Pekin ducks. *DGKD* (Diacylglycerol kinases delta) may be a common regulator of genes in co-expression networks affecting muscle and meat properties in pigs [46], and is also associated with muscle spasm in mice [47]. The down-regulated expression of *DGKD* in the *C. butyricum*-treated group may therefore also be beneficial for muscle development. *HSPB1*(heat shock protein family B member 1) is not associated with myofibrils during normal muscle development [48]. Its low expression level in the *C. butyricum*-treated group may be evidence for the increased antistress ability of the muscles in these ducks [49]. In summary, the decreased expression levels of *MYL3*, *TPM3*, *PPP1R3B*, *DGKD* and *HSPB1* in the *C. butyricum*-treated group indicate that *Clostridium butyricum* can modulate gene expression patterns to promote muscle development of Pekin ducks.

It showed that the ECM-receptor interaction, the MAPK signaling pathway and the TNF signaling pathway accounted for many of the DEGs enriched in KEGG pathway analysis. The increase in skeletal muscle yield is mainly caused by muscle hypertrophy [50]. Components of the ECM-receptor interaction contribute to the formation of the muscle niche [51]. ECM-receptor interactions are enriched in fat-tailed sheep breeds, and the level of fat deposition is a vital factor affecting meat quality. Interestingly, *LAMB4*, a gene related to the ECM-receptor interaction pathway, was also up-regulated in the tissues of fat-tailed sheep, concordantly with our results [52], suggesting that the ECM-receptor interaction may be a potent regulator of muscle cell proliferation and differentiation during skeletal muscle development. Recently, various studies have focused on the MAPK pathway in skeletal muscle development because this pathway directly affects muscle morphology by activating several myogenic transcription factors and cell differentiation [53, 54]. The DEGs between the *C. butyricum*-treated group and the control group were significantly enriched in the MAPK signaling pathway, an important regulator of skeletal muscle development. The MAPK signaling pathway can be activated during hypertrophy and facilitates increased muscle production through the increased muscle fiber size [55]. Supplementation with *C. butyricum* may therefore activate the MAPK signaling pathway and ECM-receptor interactions in the breast muscle of Pekin ducks. Further investigations are needed to explore the roles of the candidate genes in the MAPK signaling pathway and the ECM-receptor interaction in breast muscle and in determining Pekin duck meat quality. Genes that were differentially expressed between control and *C. butyricum*-treated ducks were also related to the TNF signaling pathway. This pathway may be associated with the increased immune function of ducks in the *C. butyricum*-treated group [11, 56]. Interestingly, the KEGG pathway analysis also showed enrichment of “Alanine, aspartate and glutamate metabolism”. In our previous study, supplementation with *C. butyricum* has been proved to increase the concentrations of essential amino acids and flavor amino acids in the breast muscle of ducks [11]. The enrichment of alanine, aspartate and glutamate metabolism in breast muscle provides supporting evidence for the increased meat quality of ducks in the *C. butyricum*-treated group.

To sum up, the transcriptome analysis revealed that the breast muscle yield and meat quality in *C. butyricum*-treated Pekin ducks were improved via altering the expression abundance of muscle system process genes (*MGP, FLNC, FBXO_25, LAMB4, MGAT3, MYL3, TPM3, PPP1R3B, DGKD and HSPB1*) and activating pathways associated with muscle development (mainly the ECM-receptor interaction and the MAPK signaling pathway). To obtain further information regarding the mechanism between *C. butyricum* and breast muscle yield, further investigation is demanded to optimize the effects of probiotic, especially in the livestock industry.

## Conclusion

We characterized the transcriptome profiles of breast muscle tissue in Pekin ducks supplemented with dietary *C. butyricum* by de novo assembly RNA-Seq. These differentially expressed genes may be vital for understanding the molecular mechanisms of breast muscle development induced by dietary supplementation with *C. butyricum*. GO term enrichment of the DEGs showed that regulation of muscle system processes and muscle tissue morphogenesis was significantly enriched. KEGG pathway enrichment analysis revealed that the ECM-receptor interactions, and the MAPK signaling pathway, which can promote muscle development and improve meat quality, were activated after supplementation with *C. butyricum* in Pekin ducks. To the best of our knowledge, this is the first study on the relationship between breast muscle gene expression and *C. butyricum* supplementation in Pekin ducks. It provides novel insights into the responses to dietary supplementation with *C. butyricum*, and may contribute to the optimization of the use of probiotics as feed additives for breeding duck strains with high meat yields.

## Additional files

Additional file 1: **Table S1.** Composition and nutrient level of diets (air-dry basis). **Table S2.** Quality control of each RNA sample for sequencing. **Table S3.** Evaluation of clean data of each sample in this study. **Table S4.** Effects of *Clostridium butyricum* on breast meat quality of Pekin ducks. **Table S5.** Mapping ratios of each sample to the reference duck genome in this study. **Table S6.** Mapping Statics and ratios of each sample to *de novo* assembled transcripts in this study. (DOCX 29 kb)

Additional file 2: **Figure S1.** Species distribution of the top BLAST hits. **Figure S2.** Different levels of GO term function classification. **Figure S3.** The top 20 pathways according to numbers of unigene annotation. **Figure S4.** The numbers of up- or down-regulated genes annotated to each GO term function. (DOCX 1113 kb)

Additional file 3: Detailed information of DEGs. (XLSX 135 kb)

Additional file 4: Detailed information of GO enrichment of DEGs. (XLS 31 kb)

Additional file 5: Detailed information of KEGG enrichment of DEGs. (XLSX 30 kb)

## Abbreviations

AQP1: Aquaporin 1
ASIC4: Acid sensing ion channel subunit family member 4
ATP2A: Sodium/potassium-transporting ATPase subunit alpha-2
CHRNA1: Cholinergic receptor nicotinic alpha 1 subunit
COG: Orthologous Groups of Proteins
CPS1: Carbamoyl-phosphate synthase 1
CSRP: Cysteine and glycine rich protein
ECM-receptor interaction: Extracellular matrix-receptor interaction
GO: Gene ontology
KCNC4: Potassium voltage-gated channel, Shaw-related subfamily, member 4
KCNQ5: Potassium voltage-gated channel subfamily Q member 5
MAPK: Mitogen-activated protein kinase
MYH: Myosin heavy chain, fast skeletal muscle
MYH6: Myosin heavy chain 6
MYOZ2: Myozenin 2
NCBI: National Center for Biotechnology Information
NRC: National Research Council
SCN4B: Sodium voltage-gated channel beta subunit 4
SCN5A: Sodium voltage-gated channel alpha subunit 5
TBX1: T-box-1
TNF: Tumor necrosis factor
TNNI1: Troponin I type 1 (skeletal, slow)
