# Supplementary material for: RNA-Seq transcriptome analysis of breast muscle in Pekin ducks supplemented with the dietary probiotic Clostridium butyricum
Source: BMC Genomics. 2018 Nov 28;19:844. doi: 10.1186/s12864-018-5261-1 (PMC6264624; doi:10.1186/s12864-018-5261-1)
Supplement: Supplementary file 1 — Table S1. Composition and nutrient level of diets (air-dry basis). Table S2. Quality control of each RNA sample for sequencing. Table S3. Evaluation of clean data of each sample in this study. Table S4. Effects of Clostridium butyricum on breast meat quality of Pekin ducks. Table S5. Mapping ratios of each sample to the reference duck genome in this study. Table S6. Mapping Statics and ratios of each sample to de novo assembled transcripts in this study. (DOCX 29 kb) [file 12864_2018_5261_MOESM1_ESM.docx]

Additional file 1 Tables for

RNA-Seq transcriptome analysis of breast muscle in Pekin duck supplemented with dietary probiotic *Clostridium butyricum*

Yanhan Liu^1^; Yaxiong Jia^2^; Cun Liu^2^; Limin Ding^3^*; Zhaofei Xia^1^*

^1^College of Veterinary Medicine, China Agricultural University, Beijing 100193, China

^2^Institute of Animal Sciences, Chinese Academy of Agricultural Sciences, Beijing 100193, China

^3^College of Animal Science and Technology, China Agricultural University, Beijing 100193, China

*Corresponding author:

Zhaofei Xia (Corresponding author)

Fax: +86 10 6273 3781;

E-mail address: [*zhaofeixiacau@126.com*](mailto:zhaofeixiacau@126.com)

Limin Ding (co-Corresponding author)

Fax: +86 135 0107 6373;

E-mail address: [*Dinglm2011@163.com*](mailto:Dinglm2011@163.com)

| Table S1 Composition and nutrient level of diets (air-dry basis) | | |
| --- | --- | --- |
| Item | 1-21d | 22-42d |
| Ingredients, % |  |  |
| Corn | 56.00 | 60.24 |
| Soybean meal | 32.69 | 24.67 |
| Wheat middling | 5.00 | 9.00 |
| Soybean oil | 2.10 | 1.80 |
| Phytases | 0.02 | 0.02 |
| Dicalcium phosphate | 1.00 | 1.60 |
| Limestone | 1.50 | 1.20 |
| DL-Met | 0.15 | 0.12 |
| L-Lys | 0.20 | 0.10 |
| Vitamin premix^1^ | 0.02 | 0.02 |
| Trace mineral premix^2^ | 0.20 | 0.20 |
| NaCl | 0.35 | 0.30 |
| Choline chloride (50 %) | 0.24 | 0.20 |
| Santoquin (33 %) | 0.03 | 0.03 |
| Maifanite | 0.50 | 0.50 |
| Total | 100 | 100 |
| Nutrient levels^3^,% |  |  |
| AME (MJ/kg) | 12.31 | 12.53 |
| Crude protein,% | 19.52 | 16.83 |
| Lys,% | 1.12 | 0.87 |
| Met,% | 0.46 | 0.39 |
| Calcium,% | 0.88 | 0.89 |
| Available phosphorus,% | 0.29 | 0.39 |
| Total phosphorus,% | 0.54 | 0.62 |
| Met+Cys, % | 0.79 | 0.69 |
| ^1^The vitamin premix provided the following per kilogram of diet: vitamin A, 12,500 IU; vitamin D_3_, 3500 IU; vitamin E, 20 IU; vitamin K_3_, 2.65 mg; thiamin, 2.00 mg; riboflavin, 6.00 mg; pyridoxin, 3.00 mg; VB_12_, 0.025 mg; biotin, 0.0325 mg; folic acid, 12.00 mg; pantothenic acid, 50 mg; nicotinic acid, 50.00 mg.  ^2^The mineral premix provided the following per kg of diet: Cu, 6 mg; Fe, 80 mg; Zn, 40 mg; Mn, 100 mg; Se, 0.15mg; I, 0.35 mg.  ^3^ Calculated values. | | |

| Table S2 Quality control of each RNA sample for sequencing | | | | | |
| --- | --- | --- | --- | --- | --- |
| Sample | Concentration（ng/μl） | Total（μg） | OD260/OD280 | OD260/OD230 | RIN |
| Control_A1 | 329.70 | 8.24 | 1.96 | 1.88 | 8.80 |
| Control_A2 | 719.30 | 28.77 | 2.09 | 1.78 | 9.50 |
| Control_A3 | 821.20 | 32.85 | 2.11 | 1.58 | 9.20 |
| Treatment_B1 | 637.30 | 25.49 | 2.03 | 1.82 | 9.30 |
| Treatment_B2 | 861.70 | 34.47 | 2.11 | 1.54 | 9.20 |
| Treatment_B3 | 1008.30 | 40.33 | 2.08 | 1.80 | 9.20 |

| Table S3 Evaluation of clean data of each sample in this study | | | | | | |
| --- | --- | --- | --- | --- | --- | --- |
| Sample | Total_Reads | Total_Bases | Error% | Q20% | Q30% | GC% |
| A1 | 59695558 | 8.83E+09 | 0.0123 | 98.34 | 94.91 | 55.00 |
| A2 | 64690588 | 9.57E+09 | 0.0123 | 98.32 | 94.84 | 53.92 |
| A3 | 54170296 | 8.01E+09 | 0.0126 | 98.20 | 94.52 | 55.35 |
| B1 | 54959360 | 8.14E+09 | 0.0123 | 98.31 | 94.82 | 53.95 |
| B2 | 51137356 | 7.55E+09 | 0.0124 | 98.26 | 94.67 | 55.94 |
| B3 | 61709484 | 9.13E+09 | 0.0122 | 98.38 | 94.99 | 54.66 |

| Table S4 Effects of *Clostridium butyricum* on breast meat quality of Pekin ducks | | |
| --- | --- | --- |
| Parameters | Control | Treatment |
| pH_45min_ | 6.22±0.02^a^ | 6.37±0.03^b^ |
| pH_24h_ | 5.89±0.03 | 5.90±0.03 |
| Lightness (L*) | 47.40±0.76 | 45.99±0.81 |
| Redness (a*) | 18.36±0.67^a^ | 19.86±0.71^b^ |
| Yellowness (b*) | 6.51±0.19 | 5.22±1.21 |
| Shear force (N/cm^2^) | 45.80±8.63^a^ | 31.14±3.89^b^ |
| Drip loss (%) | 10.76±1.76^a^ | 8.09±0.49^b^ |
| Values are mean ± SD of 12 independent determinations;  Means with different superscript letters indicate that there are significant differences (*P <* 0.05) between two groups in the same row;  pH_45min_: muscle pH value at 45 min postmortem; pH_24h_: muscle pH value at 24 h postmortem. | | |

| Table S5 Mapping ratios of each sample to the reference duck genome in this study | | | | | | |
| --- | --- | --- | --- | --- | --- | --- |
| type | A1 | A2 | A3 | B1 | B2 | B3 |
| Total reads | 59695558 | 64690588 | 54170296 | 54959360 | 51137356 | 61709484 |
| Total mapped | 33123642 (55.49% ) | 37239165 (57.57% ) | 28102757 (51.88% ) | 29530160 (53.73% ) | 26851566 (52.51% ) | 34387106 (55.72% ) |
| Multiple mapped | 255942 ( 0.43% ) | 279508 ( 0.43% ) | 199470 ( 0.37% ) | 160316 ( 0.29% ) | 229270 ( 0.45% ) | 262806 ( 0.43% ) |
| Uniquely mapped | 32867700 (55.06% ) | 36959657 (57.13% ) | 27903287 (51.51% ) | 29369844 (53.44% ) | 26622296 (52.06% ) | 34124300 (55.30% ) |
| Left mapped | 16713419 (28.00% ) | 18810342 (29.08% ) | 14210854 (26.23% ) | 14916835 (27.14% ) | 13569246 (26.53% ) | 17349511 (28.11% ) |
| Right mapped | 16410223 (27.49% ) | 18428823 (28.49% ) | 13891903 (25.64% ) | 14613325 (26.59% ) | 13282320 (25.97% ) | 17037595 (27.61% ) |

| Table S6 Mapping Statics and ratios of each sample to *de novo* assembled transcripts in this study | | | | | | | | |
| --- | --- | --- | --- | --- | --- | --- | --- | --- |
| Sample | Total reads | Total mapped | Multiple mapped | Uniquely mapped | Reads mapped to '+' | Reads mapped to '-' | Read-1 | Read-2 |
| A1 | 59695558 | 51698314  (86.60%) | 11018024  (18.46%) | 40680290  (68.15%) | 20340145  (34.07%) | 20340145  (34.07%) | 20340145  (34.07%) | 20340145  (34.07%) |
| A2 | 64690588 | 56863042  (87.90%) | 11811980  (18.26%) | 45051062  (69.64%) | 22525531  (34.82%) | 22525531  (34.82%) | 22525531  (34.82%) | 22525531  (34.82%) |
| A3 | 54170296 | 47906218  (88.44%) | 8843328  (16.33%) | 39062890  (72.11%) | 19531445  (36.06%) | 19531445  (36.06%) | 19531445  (36.06%) | 19531445  (36.06%) |
| B1 | 54959360 | 48284460  (87.85%) | 9301020  (16.92%) | 38983440  (70.93%) | 19491720  (35.47%) | 19491720  (35.47%) | 19491720  (35.47%) | 19491720  (35.47%) |
| B2 | 51137356 | 45259894  (88.51%) | 7940796  (15.53%) | 37319098  (72.98%) | 18659549  (36.49%) | 18659549  (36.49%) | 18659549  (36.49%) | 18659549  (36.49%) |
| B3 | 61709484 | 54291950  (87.98%) | 10563548  (17.12%) | 43728402  (70.86%) | 21864201  (35.43%) | 21864201  (35.43%) | 21864201  (35.43%) | 21864201  (35.43%) |
