# Supplementary material for: RNA-Seq transcriptome analysis of breast muscle in Pekin ducks supplemented with the dietary probiotic Clostridium butyricum
Source: BMC Genomics. 2018 Nov 28;19:844. doi: 10.1186/s12864-018-5261-1 (PMC6264624; doi:10.1186/s12864-018-5261-1)
Supplement: Supplementary file 2 — Figure S1. Species distribution of the top BLAST hits. Figure S2. Different levels of GO term function classification. Figure S3. The top 20 pathways according to numbers of unigene annotation. Figure S4. The numbers of up- or down-regulated genes annotated to each GO term function. (DOCX 1113 kb) [file 12864_2018_5261_MOESM2_ESM.docx]

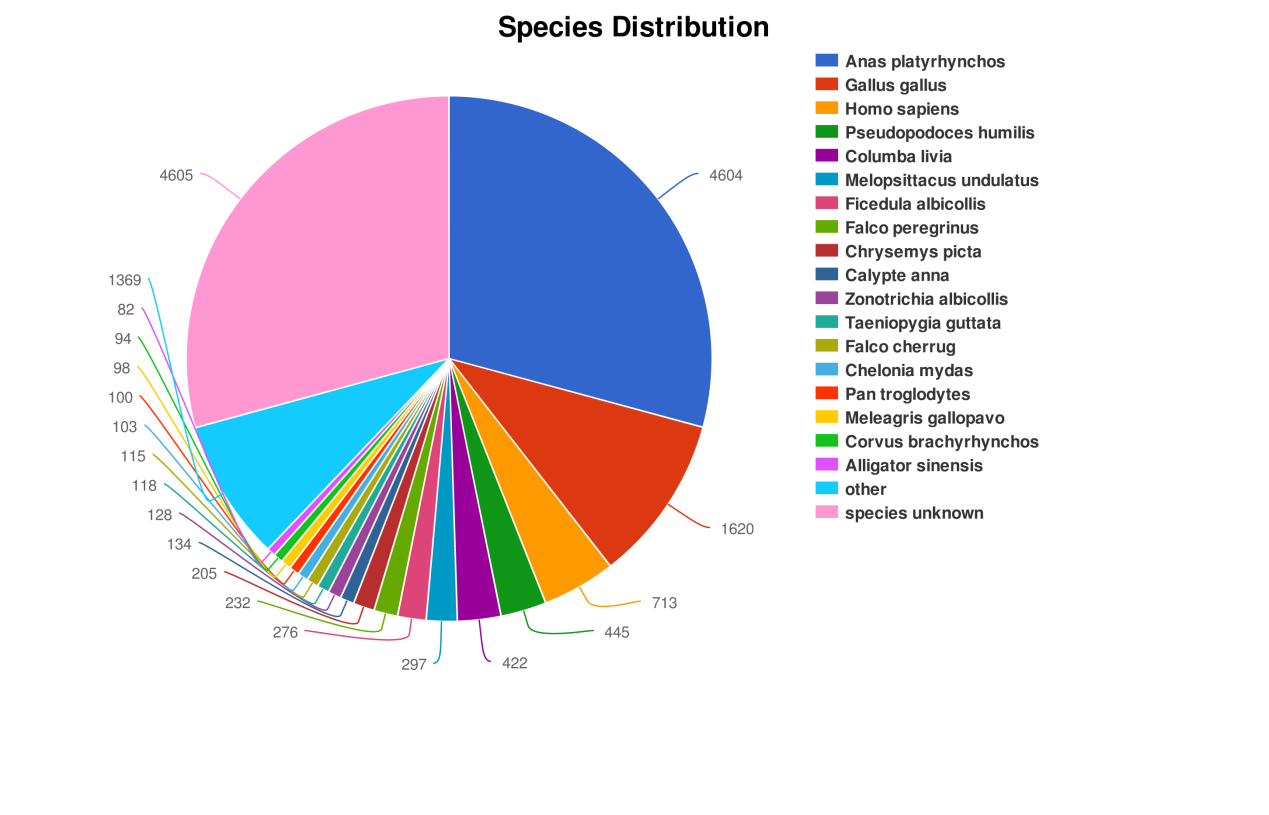


Figure S1 Species distribution of the top BLAST hits. Homology search for the sequences obtained after clustering was done using BLASTX against protein sequences in the NCBI NR databases. The maximum homology species of Pekin duck transcripts were *Anas platyrhynchos.*


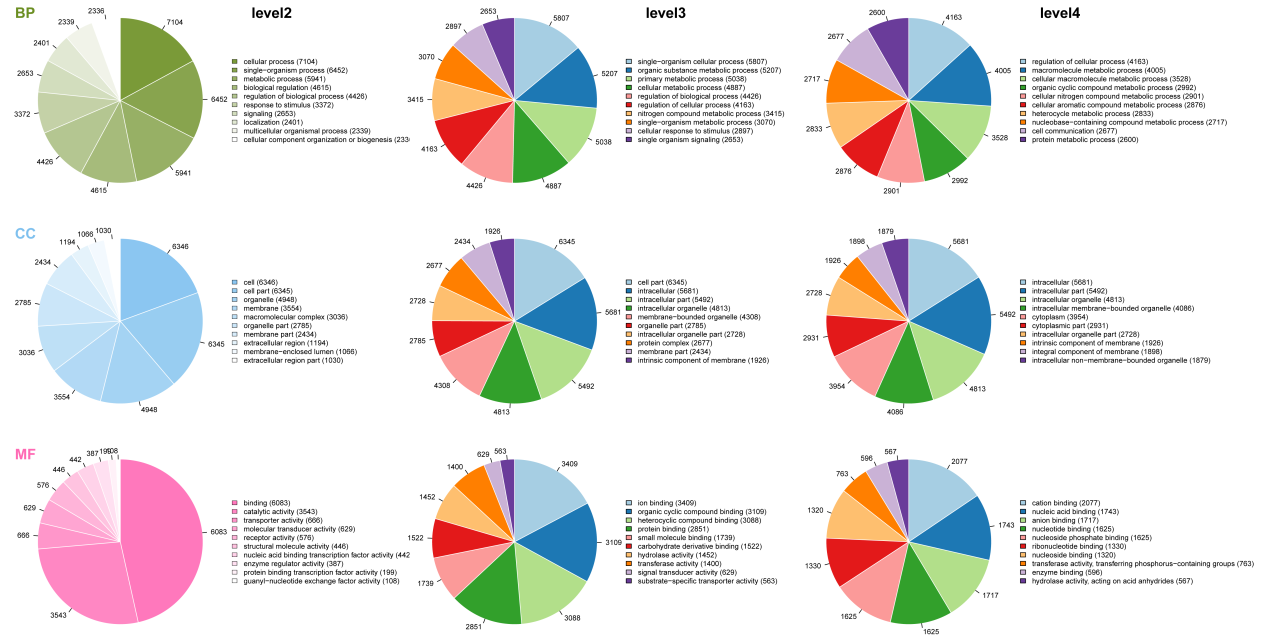
Figure S2 Different levels of GO term function classification. BP, biological process; CC, cellular component; MF, molecular function.


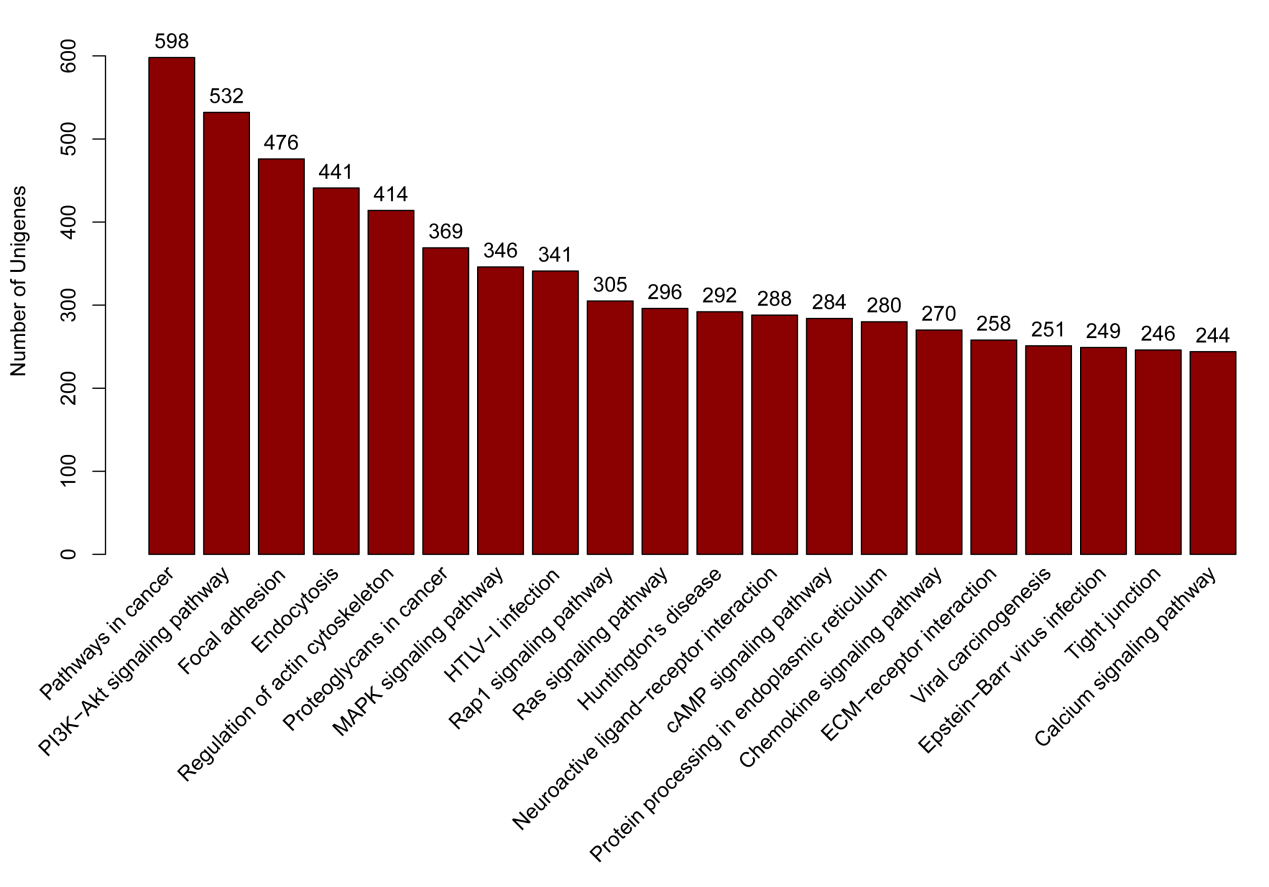


Figure S3 The top 20 pathways according to numbers of unigene annotation.


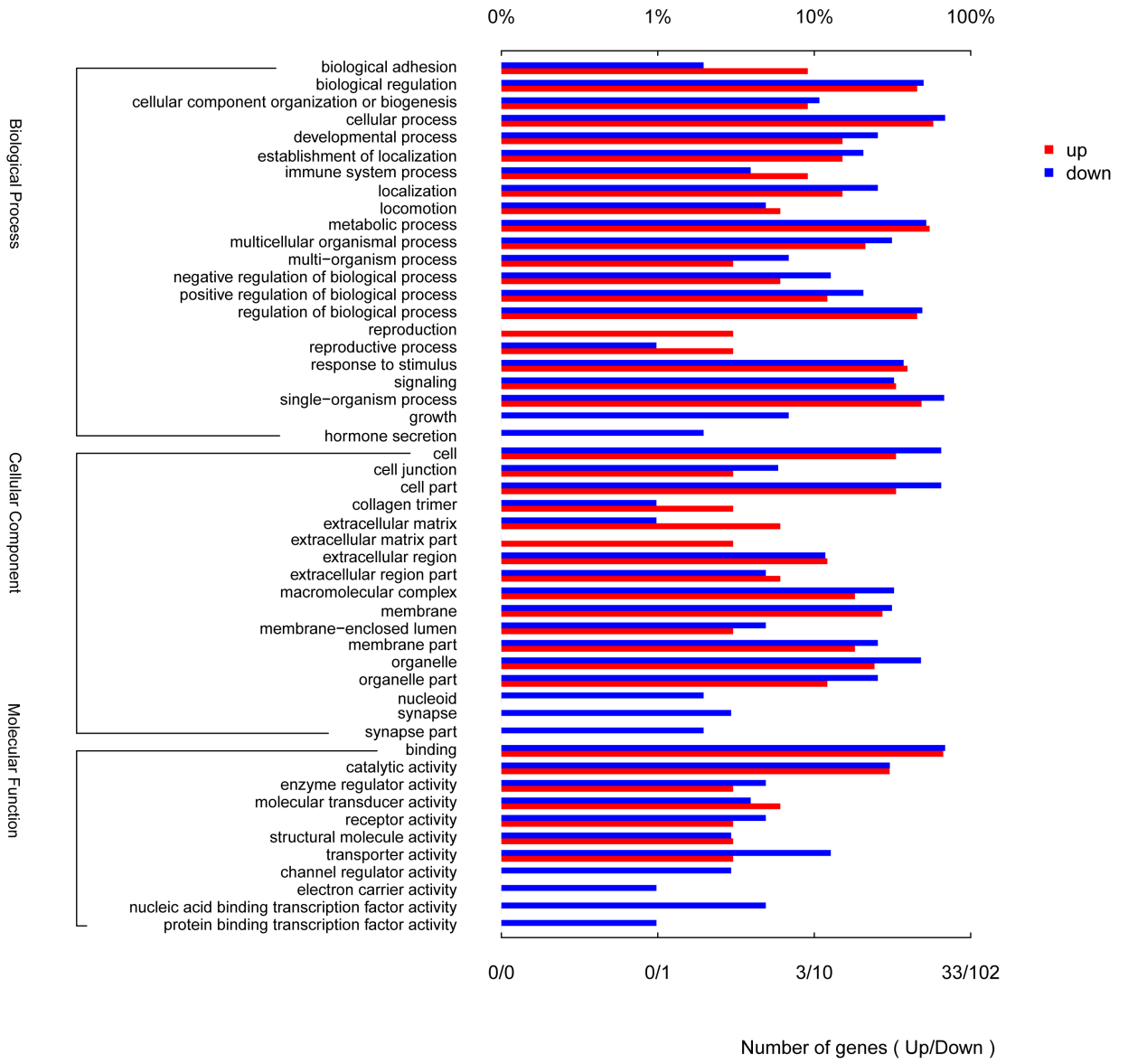


Figure S4 The numbers of up- or down-regulated genes annotated to each GO term function.
